# Monitoring Hardening Behavior of Cementitious Materials Using Contactless Ultrasonic Method

**DOI:** 10.3390/s21103421

**Published:** 2021-05-14

**Authors:** Jinyoung Hong, Hajin Choi

**Affiliations:** School of Architecture, Soongsil University, Seoul 06978, Korea; jinyoung23@soongsil.ac.kr

**Keywords:** contactless ultrasonics, sensor array, rapid setting cement, setting, hardening

## Abstract

We propose a novel contactless ultrasonic method for monitoring the hardening behavior of cementitious materials. The goal of this method is to obtain high-quality data to compare the unique hardening process between rapid setting cement (RSC) and ordinary Portland cement (OPC) mortars without physical coupling to the surface of the specimens. To monitor the hardening behavior of cementitious materials, conventional approaches use contact or embedded-type sensors, which limit field application. Our solution is to measure leaky Rayleigh waves at the interface between air and cementitious materials, which allows for the estimation of the physical state of the medium in real time. The modulus development was back-calculated based on the increment of wave velocity using the developed sensor array and transform-based signal processing. We experimentally demonstrated that the proposed method possibly exhibits unique hardening information about flash setting, effects of a retarder, and modulus increments from RSC specimens.

## 1. Introduction

The hardening behavior of cementitious materials, such as setting, is an important characteristic in construction sites. The setting is generally considered a time-zero point between liquid and solid states. Overall workflow in construction is scheduled depending on the setting of cementitious materials. Despite its practical importance, the standard definition of setting is arbitrary. Based on four standards (ASTM C 191, 266, 403, and 953), empirical values based on penetration tests are used to define the setting of cementitious materials [1,2,3,4]. The times determined for the measures in these standards are not identical [5]. Furthermore, penetrating test methods are not directly applicable to fresh concrete, owing to misleading values caused by coarse aggregates. Therefore, a mortar fraction should be obtained by labor-intensive wet sieving to remove coarse aggregates from the paste. However, the hardening behavior of the mortar fraction is not identical to that of concrete, owing to the different compositions of the materials [6].

Ultrasonic testing methods have been applied to monitor the hardening behavior of cementitious materials [7,8]. As ultrasonic wave propagation is directly governed by the physical properties of the medium, there is an advantage to estimating the hardening status of a medium. In particular, the characteristics of shear waves have been used to define the setting because shear resistance is a unique feature of solids, and shear wave propagation is governed by the shear modulus of the medium. Shear wave reflection (ultrasonic wave reflection) has been investigated to monitor the hydration of cementitious materials [9,10,11,12,13], in which an applied shear wave transducer is attached to the buffer next to the materials to measure the amount of wave reflection. Further investigation was performed by Zhu et al. [14] and Liu et al. [15], in which embedded transducers were used to measure shear waves, and the increase in shear wave velocity exhibited a good correlation with concrete hardening and setting.

A recently developed contactless ultrasonic method has significantly improved the practical application of monitoring the hardening of fresh concrete [16,17]. In this method, the leakage portion of Rayleigh waves is measured, in which the motion of Rayleigh waves is between shear and pressure waves. Similar to shear waves, Rayleigh wave propagation is also a unique characteristic of solids. In a joint half-space, such as air and concrete, leaky Rayleigh waves propagate at the interface between air and concrete. Therefore, the waves can be measured in air without physical coupling to the concrete. In this case, the contactless ultrasonic method can be used to monitor the hardening behavior of concrete. This method does not require the pre-installation of sensors inside the concrete or the surface coupling procedure, enabling the acquisition of information of fresh concrete in early stages, without any preparation. Choi et al. [16] experimentally demonstrated that the contactless ultrasonic method is applicable to cement paste, mortar, and concrete. Hong et al. [17] indicated that the definition of the time-zero point by the contactless ultrasonic method is more accurate compared to ASTM C 403 under accelerated curing.

In this study, we present further improvements to the contactless ultrasonic method in the sensing system and its analysis. First, the hardware is improved using an array of sensors along the traveling wave path. Second, the phase difference between the measured waves through the array can be calculated. Therefore, the velocity of the leaky Rayleigh wave can be accurately estimated, and the corresponding dynamic modulus of the fresh concrete is calculated. This study includes the following unprecedented contributions: (1) the contactless method was applied to monitor the modulus development of rapid setting cement (RSC) and the results were compared with those of ordinary Portland cement (OPC), and (2) the velocity of leaky Rayleigh waves was calculated based on phase differences among propagating waves using a two-dimensional Fourier transform. Specifically, the details of amplifying-circuit design for sensor arrays and its application are presented. This article includes a brief description of the contactless ultrasonic method (Section 2), experimental validation including testing materials and hardware improvement (Section 3), and signal processing and dynamic modulus monitoring techniques (Section 4). We further discuss the possible estimation of the static modulus using the contactless ultrasonic method in Section 5.

## 2. Methodology

Rayleigh waves propagate along the surface when the pressure and shear waves are guided at the boundary. In the case of joint-half space (liquid and solid), a portion of Rayleigh waves is leaked into the liquid, which is called leaky Rayleigh waves. The characteristic equations of Rayleigh waves in the joint-half space are presented as follows:

(1) $$4k^{2}qs-{\left(k^{2}+s^{2}\right)}^{2}=i\frac{\rho_{L}}{\rho }\frac{qk_{s}^{4}}{\sqrt{k_{L}^{2}-k^{2}}}$$

(2) $$q^{2}=k^{2}-k_{p}^{2},$$

(3) $$s^{2}=k^{2}-k_{s}^{2}$$

where $k_{p}$ and $k_{s}$ are the pressure and shear wave number in the solid, respectively, k is the Rayleigh wave number, and $\rho_{L}$ and ρ are the densities of the liquid and solid, respectively. In general, the Rayleigh wave number has complex roots, where the imaginary part of the solution represents the leakage of waves into the liquid. Therefore, leaky waves occur on the liquid side, whereas the characteristics of the waves are related to the solid medium.

For air and concrete, the density ratio between $\rho_{L}$ and ρ is approximately 0.0005, with values of 1.225 and 2400 kg/m^3^ for air and concrete, respectively. With the minimal ratio, the velocity difference between Rayleigh and leaky Rayleigh waves is less than 0.5% [18]. Therefore, the relation between the Rayleigh wave velocity and material properties can be used in the case of air and concrete. In this study, the velocity of leaky Rayleigh waves measured in air was used to calculate the dynamic modulus of concrete without losses. The dynamic modulus of concrete can be back-calculated based on Equation (4):

(4) $$V_{R}=\frac{0.87+1.12\upsilon }{1+\upsilon }\sqrt{\frac{E_{d}}{2\rho \left(1+\upsilon \right)}}$$

where υ and $E_{d}$ are the Poisson’s ratio and the dynamic modulus of elasticity in concrete, respectively.

In this study, we employed a contactless ultrasonic method to monitor the hardening behavior of concrete. This case can be considered as wave propagation in air and quasi-state–solid. As the state of cementitious materials varies from liquid to solid, leaky Rayleigh waves are not measurable at the beginning because of the lack of shear resistance. When the state of the medium becomes solid (considered as time-zero), leaky Rayleigh waves are propagated at the interface. The initiation of leaky Rayleigh waves is an important factor in this study, as it corresponds to the setting of cementitious materials. As the hardening continues, the velocity of the waves increases accordingly. Therefore, the increment in the velocity directly represents the modulus development after setting.

In the experiment, the incident angle of ultrasound should be carefully designed to excite Rayleigh waves at the interface between the air and fresh concrete. Based on Snell’s law, the velocity ratio influences the critical angle, and the velocity in cementitious materials is a function of time in this case. Choi et al. and Hong et al. experimentally demonstrated that the contactless ultrasonic method measured initial leaky Rayleigh waves with an incident angle of 5°, and the time was close to the final setting based on ASTM C 403 [3,16,17]. Therefore, an incident angle of 5° was chosen for the entire testing operation in this study.

**Figure 1:**
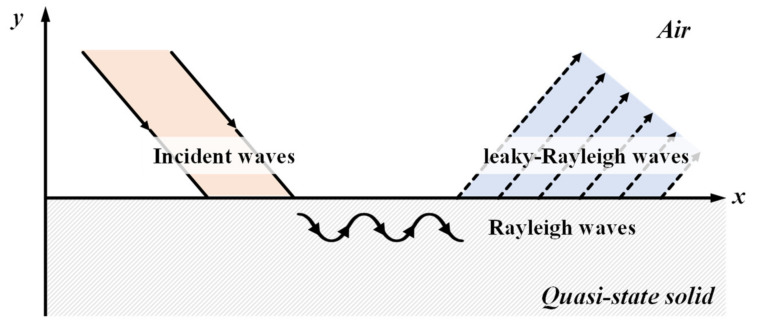
Schematic illustration of the leaky-Rayleigh waves and sensor array methodology.

**Figure 2:**
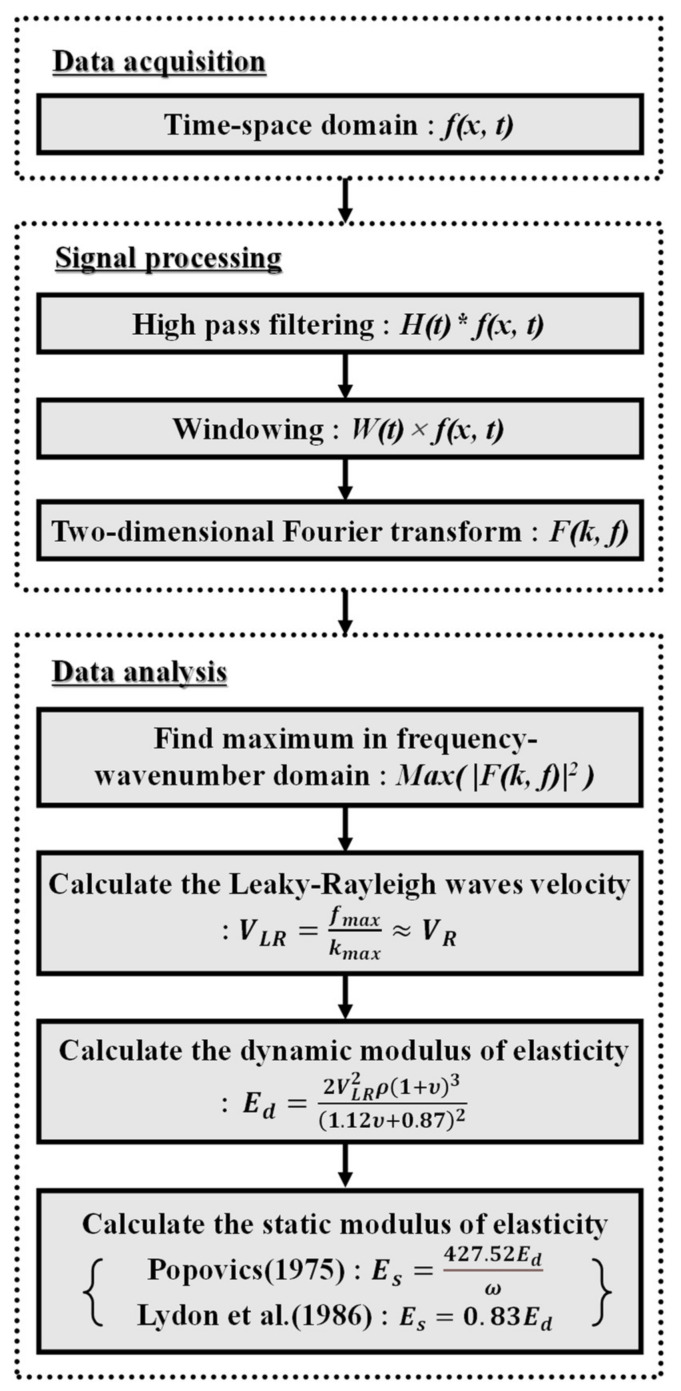
Flowchart of proposed method.

After setting, the hardening behavior of the cementitious materials can be estimated based on the velocity increment. In this study, the sensor array enabled the measurement of waves along the travel. With regular spacing among the sensors, the velocity of the propagating waves can be calculated based on the phase difference between the sets of waves. Two-dimensional Fourier transforms were applied to the set of waves in the time-space domain. Then, the highest amplitude was used to calculate the velocity at the moment in the frequency-wave number domain. The transform-based approach possibly minimizes the error in the velocity calculation among the discrete time series. A schematic illustration of the data acquisition and the procedure of hardening behavior monitoring are shown in Figure 1 and Figure 2, respectively.

## 3. Experimental Validation

### 3.1. Materials and Test Specimens

Two different cementitious materials were used in the study. RSC is mainly composed of alumina (C_11_A_7_ and CaF_2_), amorphous alumina (C_12_A_7_ and CaSO_4_), and haüyne (C_3_A_3_·CaSo_4_). RSC has been used as a repair material in the field because of its rapid strength development. For reference, OPC was also used, and a detailed composition of the materials is presented in Table 1. RSC contains a large amount of 3CaO, 3Al_2_O_3_, and CaSO_4_ compared to OPC, which provides the fast hardening during the hydration reaction [5].

The contactless ultrasonic method was experimentally evaluated with four different mortar specimens: three with RSC and one with OPC. The detailed mixing ratios of the mortar specimens are summarized in Table 2. The same water-to-cement (W/C) ratio of 50% was used for all specimens. The differences among specimens with RSC were related to the use and dosage of chemical admixtures, specifically a retarder. The applied dosages of the retarder in the RSC specimens were 0%, 0.2%, and 0.4% of the total cement volume. Therefore, different setting behaviors were expected for the specimens produced. In the preparation of mortars, the fine aggregate was first mixed with cement and the retarder was dissolved in water. The materials were mixed for 3 min after the addition of water, and the mortar was immediately poured in 400 × 100 × 100 mm beam-type molds.

### 3.2. Hardware Development and Testing Set-Up

**Figure 3:**
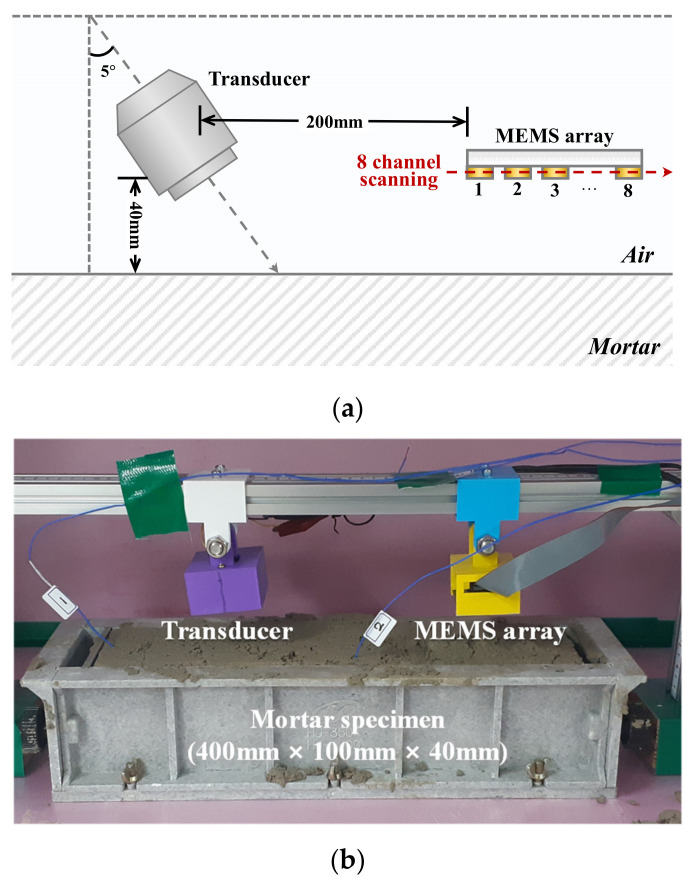
Testing setup of contactless ultrasonic method: (**a**) conceptual diagram, (**b**) photo of experimental setup.

**Figure 4:**
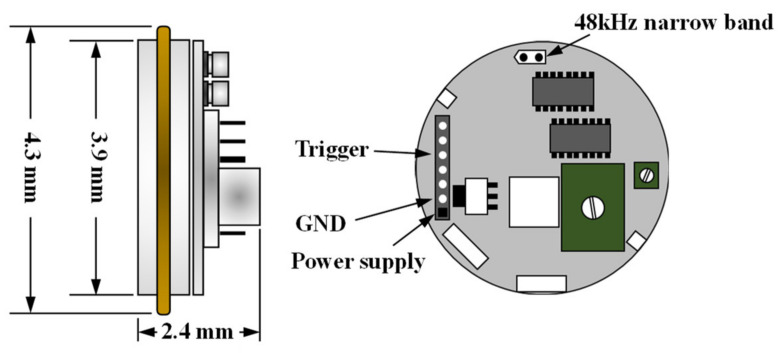
Details of the contactless ultrasonic transducer [19].

The testing setup for the contactless ultrasonic method is shown in Figure 3. A fully non-contact testing scheme was applied to the liquid state of the mortar specimens, where the lift-off distance was set to 40 mm. For the transmitter, an air-coupled ultrasonic transducer (PID-615089, SensComp) was used, which generated 16 cycles of sinusoidal signals, with a narrow band at a center frequency of 48 kHz [19]. The incident angle was set to 5°, which is the critical angle for wave velocities of the specimen and air of 3900 and 343 m/s, respectively. A detailed illustration of the transducer is shown in Figure 4.

**Figure 5:**
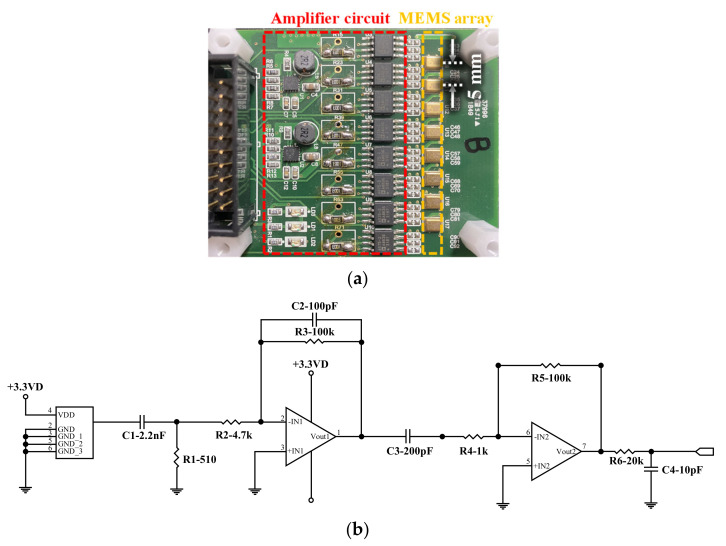
Details of developed sensor array: (**a**) 8 ch-MEMS array, (**b**) designed amplifying circuit.

In the case of receivers, a total of 8 channels of micro-electromechanical system (MEMS) were aligned with linear intervals of 5 mm, as shown in Figure 5a. The MEMS (SPU0410LR5H-QB, Knowles Acoustics) exhibit a frequency response with a 1–10 dB response gain in the audible frequency range of 10–20 kHz, and an −8 dB response gain in the ultrasonic range above 35 kHz. It is noted that each sensor price is USD 0.5 on the market. To increase the sensitivity of the signals at 48 kHz, an amplifying circuit was designed, as shown in Figure 5b, whose detailed values of resistance and capacitance are listed in Table 3. It is noted that the main frequency was chosen for concrete application to avoid scattering effect due to coarse aggregate. Signal collection starts when the four pins connected to the MEMS have a 3.3-V_DD_ potential difference based on the ground (GND). The collected signals primarily pass through a resistor-capacitor (RC) filter. The RC filter includes a capacitor and resistance in the serial and parallel connections, respectively. The signal passing through the RC filter generates a signal according to Kirchhoff’s equation, as expressed by Equation (5):

(5) $$V_{\mathrm{out}}=\frac{R_{1}}{\sqrt{X_{c}{}^{2}+R_{1}{}^{2}}}V_{\mathrm{in}}$$

(6) $$X_{c}=\frac{1}{\omega c}$$

where $V_{\mathrm{out}}$ is the output voltage, $X_{c}$ is the capacitor reactance, $R_{1}$ is the resistance, $V_{\mathrm{in}}$ is the input voltage, ω is the angular frequency, and c is the capacitance of the capacitor. The capacitance has a value of 2.2 nF and a reactance of 1.45. kΩ for 50 kHz, based on Equation (5), whereas the resistance has a value of 510 Ω. The filter acts as a high-pass filter because output voltage decreases as input frequency decreases.

**Figure 6:**
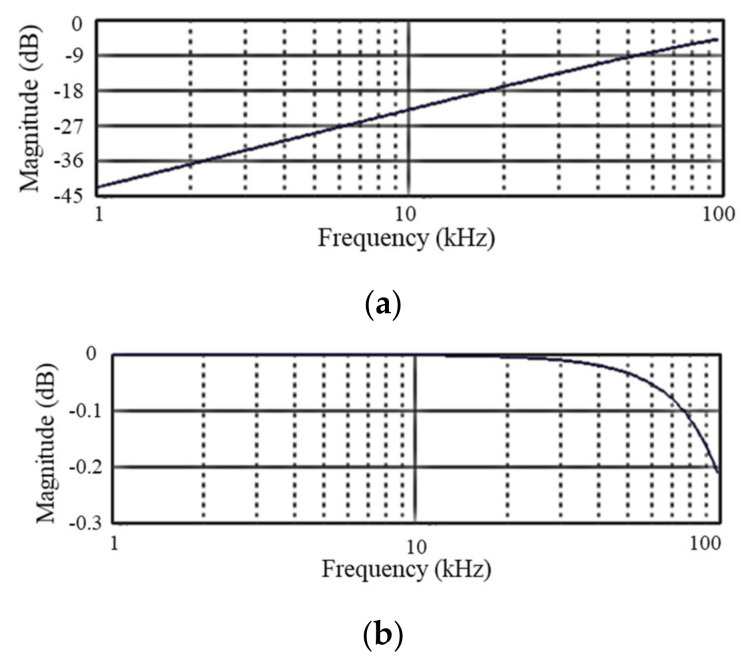

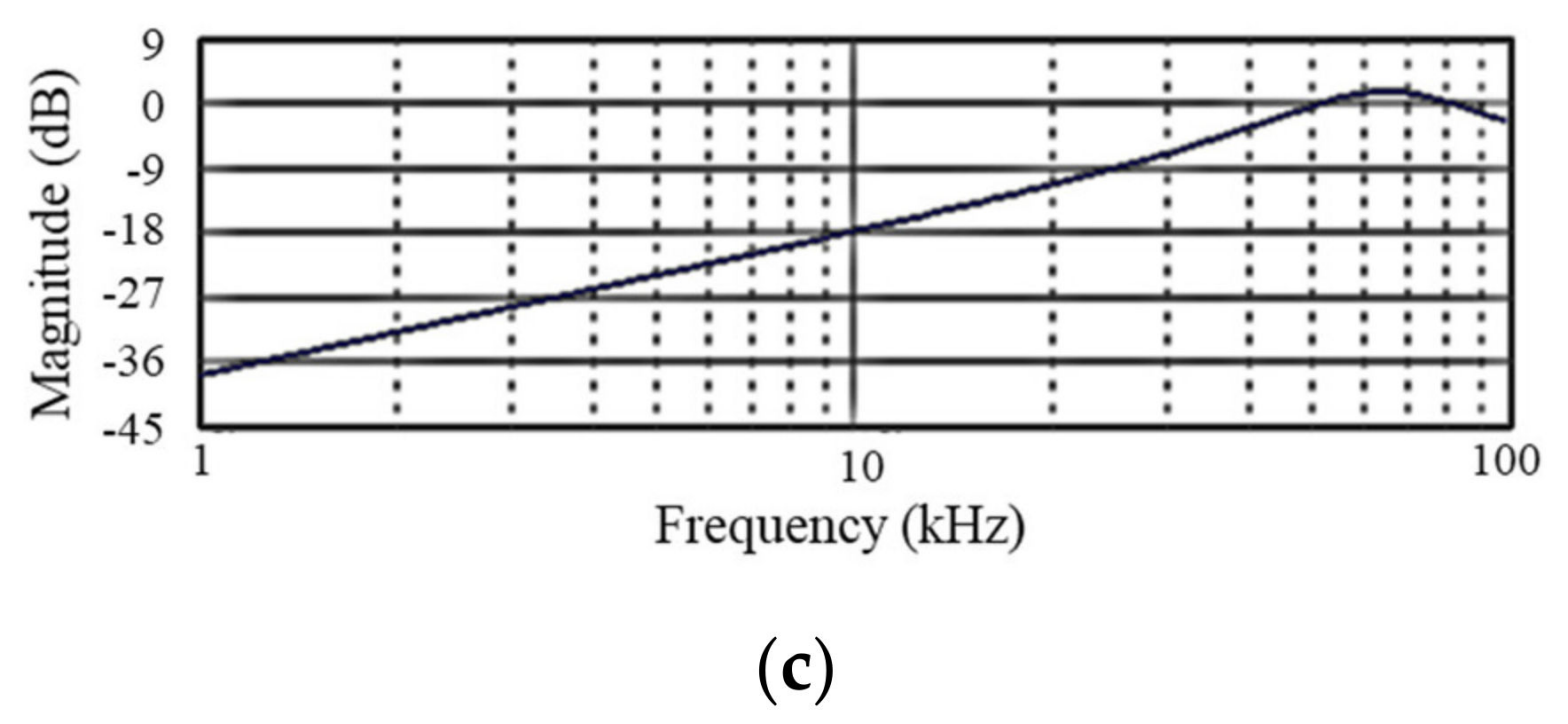
Filter performance results: (**a**) 1st filter; (**b**) 2nd filter; (**c**) total filters.

The output voltage through the RC filter is amplified by an operational amplifier (OP-AMP), wherein the phase of the input signal is reversed. The input signal through the amplifier is governed by Equation (7), and the relationship between $X_{c}$ and $R_{3}$ can be defined as shown in Equation (8).

(7) $$V_{\mathrm{out}}=-\frac{X_{C}∥R_{3}}{R_{2}}V_{\mathrm{in}}=-\frac{R_{3}}{R_{2}}\frac{1}{\left(1+2\pi fC_{2}\right)}V_{\mathrm{in}}$$

(8) $$(X_{C}∥R_{3})=\frac{1}{\frac{1}{X_{c}}+\frac{1}{R_{3}}}$$

where $R_{2}$ and $R_{3}$ represent the resistances connected in series and parallel, respectively. The $R_{2}$ and $R_{3}$ used in the circuit are 4.7 kΩ and 100 kΩ, and the corresponding output voltage becomes approximately 20 times the input voltage. In addition to amplification, the circuit acts as a low-pass filter because voltage increases as frequency decreases. As signals through the OP-AMP may generate a low-frequency direct current (DC) offset, an additional capacitor was installed to remove this. Ultimately, the signals passing through the electrical circuit exhibited the highest sensitivity at 50 kHz and were amplified approximately 2000 times. The phase of the signals is the same as that of the initial phase using two inverting amplifiers. The sensitivity of each filter design in frequency domain is presented in Figure 6.

The distance between the transducer and the first receiver was set to 200 mm. The obtained signal was digitized using NI-DAQ 6366 with 2000 samples and a sampling rate of 2 MHz. The signal was time-averaged 100 times, and the averaged signal was stored every 10 min The total duration of the experiment per specimen was 24 h.

## 4. Results

### 4.1. Results of the Contactless Ultrasonic Method Based on Single Sensor

**Figure 7:**
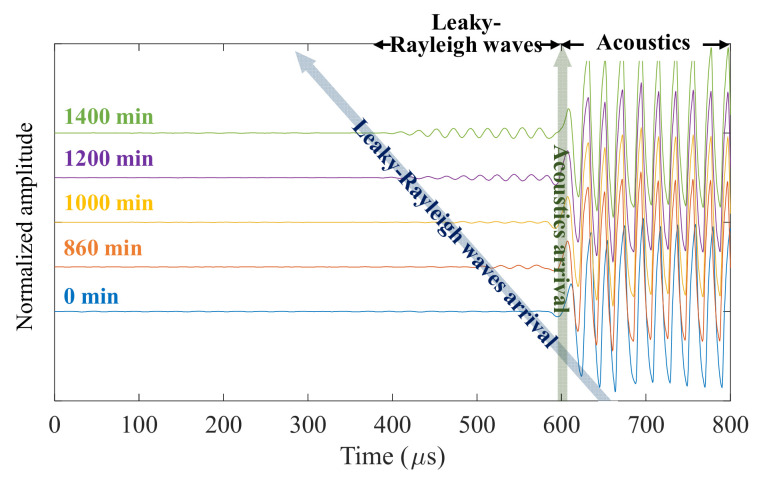
Obtained A-scan signals by the single sensor presenting development of leaky Rayleigh waves from OPC 0%.

Leaky Rayleigh waves were measured in mortar specimens using the contactless ultrasonic method. As described in Section 2 (joint-half space between liquid and quasi-solid), the leaky waves are not measurable at the beginning of hydration. Thus, the waves were initiated at a certain time when the mortar became solid. The example time signals from OPC 0% are presented in Figure 7, where a series of A-scans were obtained from the first sensor among the arrays. The initiation of leaky Rayleigh waves was approximately 800 min after the addition of water. Then, the arrivals of the leaky waves increased as the mortar hardened. The developed amplifying circuit and sensor array clearly captured the leakage portion of Rayleigh waves from the quasi-state solid, despite the inherently low amplitude. In contrast to leaky Rayleigh waves, the direct acoustics between the transducer and sensor exhibited a large amplitude with stable arrivals. This is because there was no variance in the air during the operation of the applied method.

**Figure 8:**
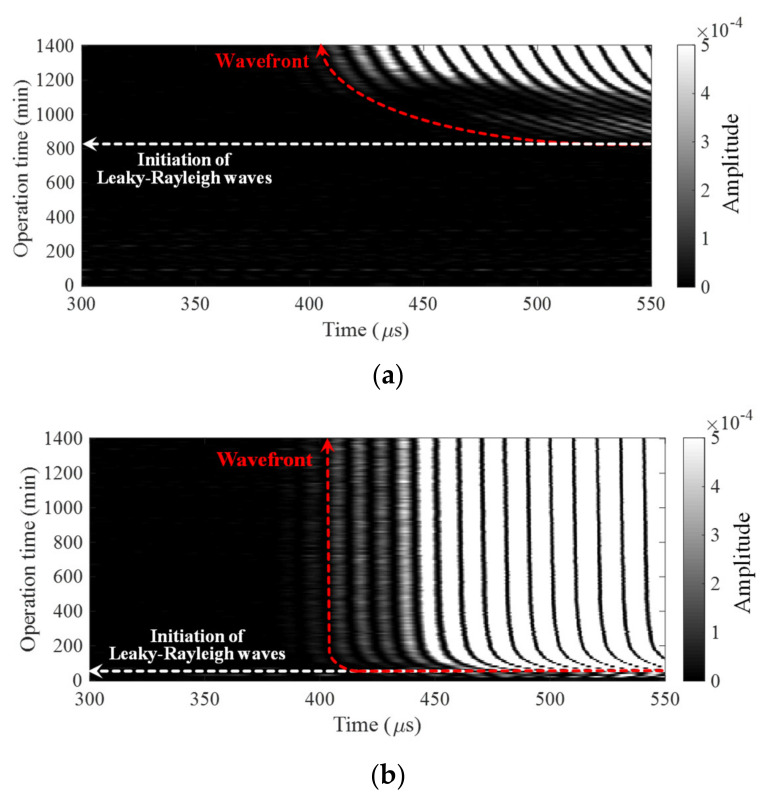

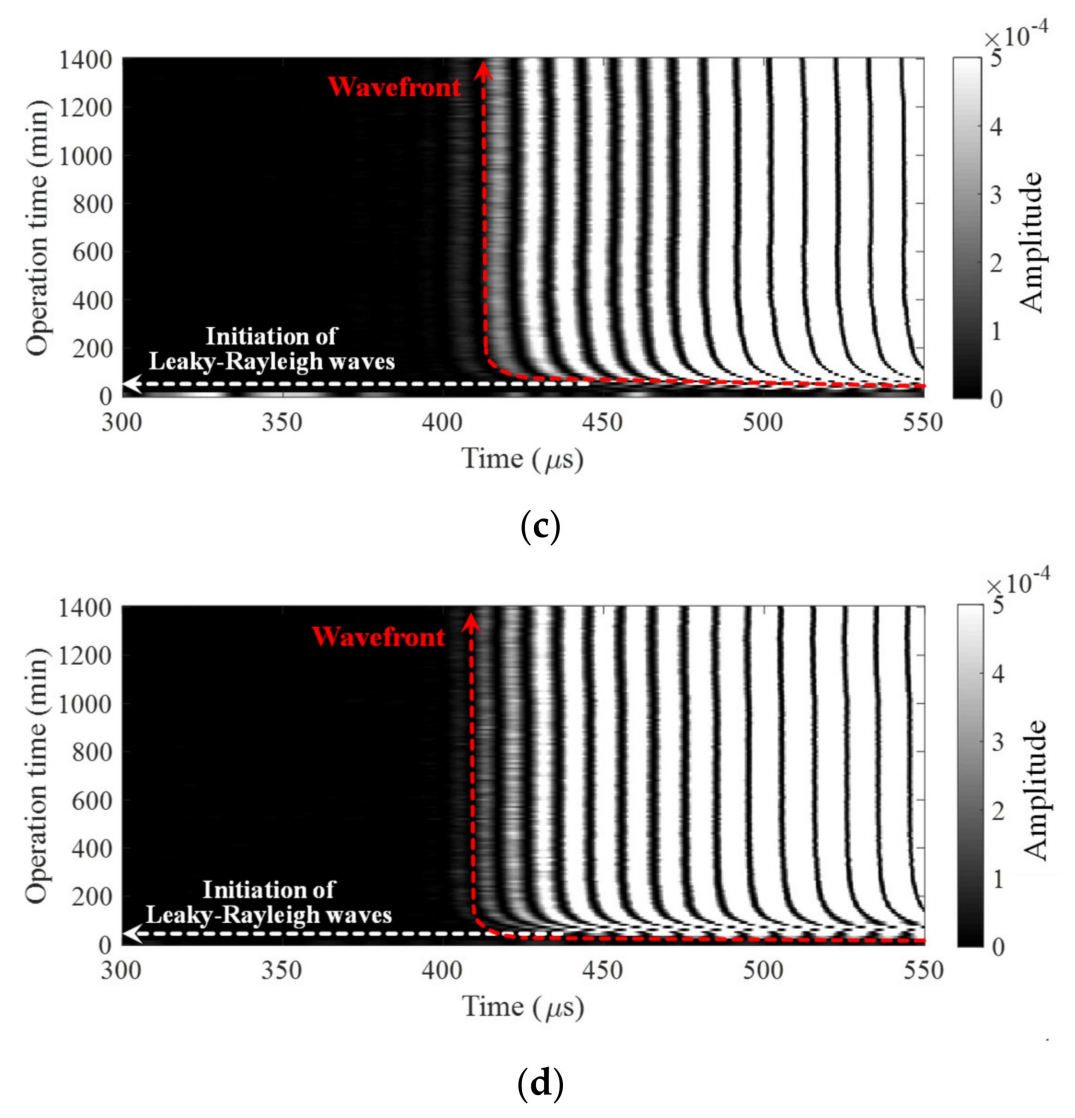
Visualization of leaky Rayleigh wave arrivals, measured by the single sensor showing the initiation of the waves and wavefront increment: (**a**) mix OPC 0%, (**b**) mix RSC 0%, (**c**) mix RSC 0.2%, (**d**) mix RSC 0.4%.

The set of obtained signals by the first sensor among the array during the hydration reaction (called operation time in the vertical axis) is visualized in Figure 8. The images clearly show the initiation of leaky Rayleigh waves and the increment of the wavefront during the operation time. On top of the images, the initiation and the increment are marked by white and red dotted lines, respectively. The two important parameters were easily obtained by a 25% amplitude difference between the noise level and oscillation. Using simple visualization of the data, the setting and hardening behaviors of cementitious materials can be identified.

When comparing OPC 0% and RSC 0%, the initiation of leaky waves was 860 and 20 min, respectively, which corresponds to the final setting, defined by ASTM C 403. From the RSC 0%, leaky Rayleigh waves were measurable at very early ages. Such characteristics are due to the large amounts of 3CaO, 3Al_2_O3, and CaSO_4_. After initiation, the arrival of the wavefront represents the hardening behavior of the cementitious materials. In the case of RSC 0%, the arrival of the wavefront was exponentially developed, and converged at a certain period. However, the arrival of the wavefront in OPC 0% presented a slow progress over the operation time compared to RSC 0%. Based on the unique behavior of the wavefront increment in RSC 0%, it is known that the hardening progress is extremely fast, and the hydration reaction is almost complete after setting. When the retarder was added to the RSC specimens, the initiation of leaky Rayleigh waves was delayed by 10 min for both RSC 0.2% and RSC 0.4%. However, the delay was minimal based on the experimental setup, in which the measurements were performed every 10 min. In addition to the initiation, the wavefront increment from both specimens with the retarder was not distinct from that of RSC 0%.

### 4.2. Signal Processing for Velocity of Leaky Rayleigh Waves Based on Sensor Array

**Figure 9:**
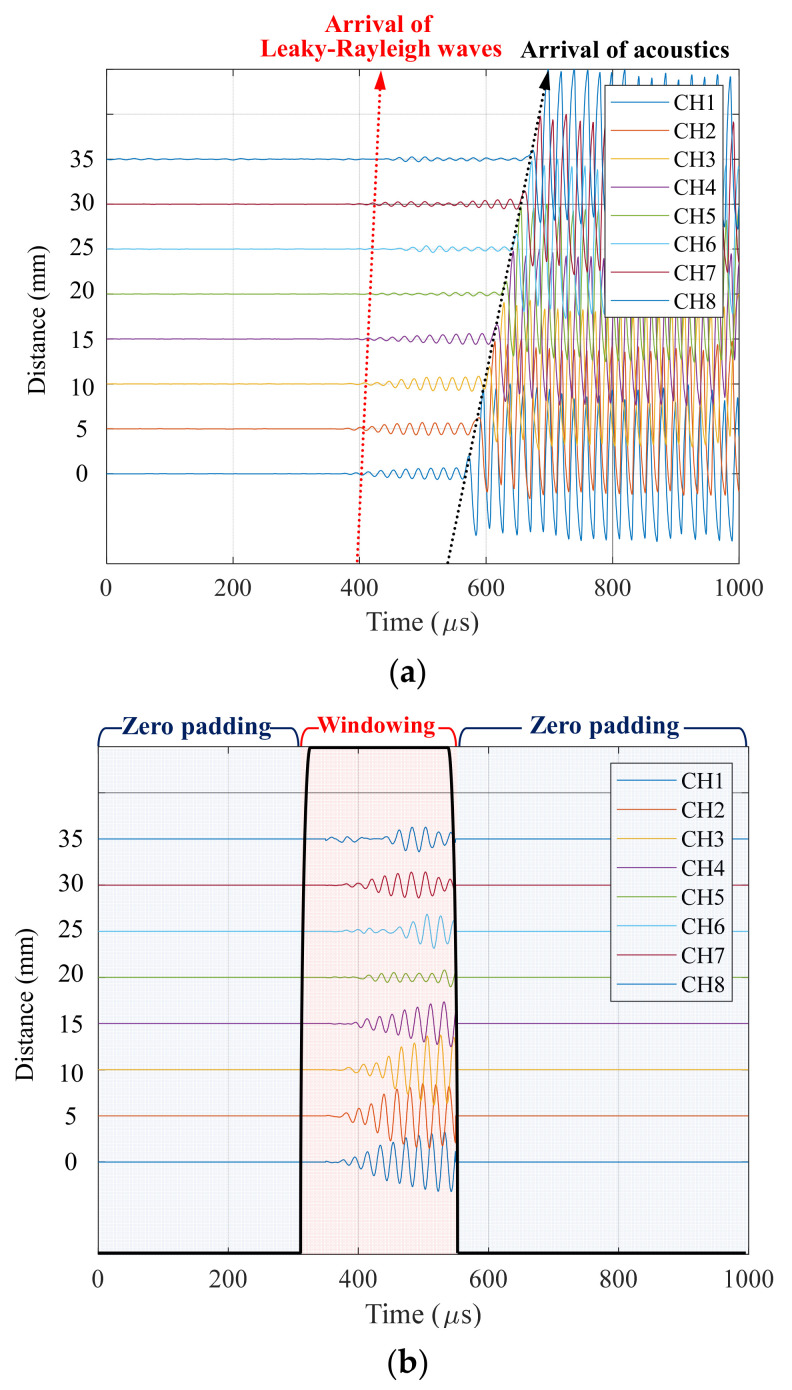
Set of signals obtained by the sensor array: (**a**) example data in time-space domain, (**b**) example of windowed signals to extract a leakage portion.

The developed sensor array provides comprehensive information on wave propagation during hardening behavior in cementitious materials, which overcomes the limitations of experimental interpretation by a single sensor. In the example data, the value of $f\left(x,t\right)$ measured by the sensor array is shown in Figure 9a, presenting the different arrivals of leaky Rayleigh waves and direct acoustics. The slope of the arrival represents wave velocity. As the hardening process in mortars continues, the slope from leaky Rayleigh waves keeps increasing, whereas that from acoustics remains stable. However, the slope of leaky Rayleigh waves was difficult to identify accurately because of its small amplitude. Furthermore, the arrival peaking from discrete data is a time-consuming procedure, as well as a subjective definition by the operator.

**Figure 10:**
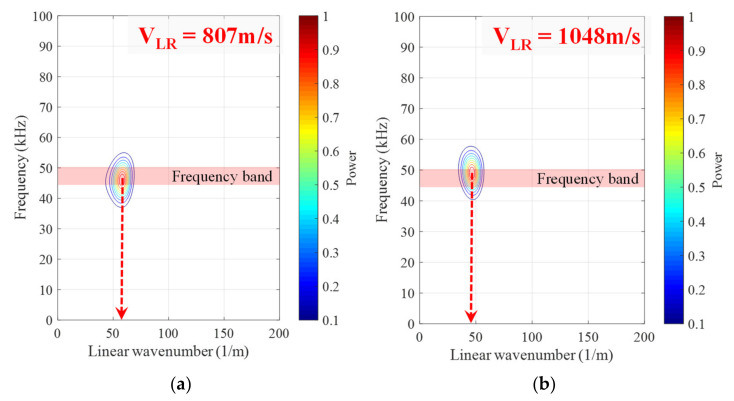

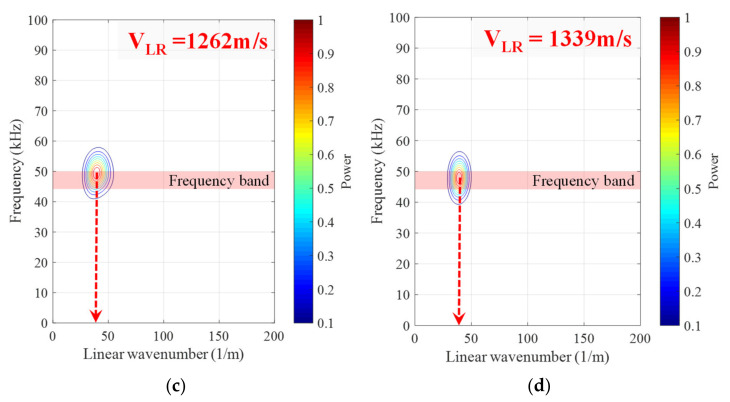
Examples of frequency-wave number domain analysis at different states of OPC 0%, showing operation time at (**a**) 860 min, (**b**) 1000 min, (**c**) 1200 min, (**d**) 1400 min.

To improve the accuracy of data analysis and extract velocity information objectively, the phase difference of eight signals was calculated in the frequency-wave number domain. After high-pass filtering, only the leakage portion was extracted using the Tukey windowing function. Zeros were added to maintain the same length as the original data, as presented in Figure 9b. Then, a two-dimensional Fourier transform was applied to the data as follows:

(9) $$F\left(k,\omega \right)=\iint f\left(x,t\right)e^{-i2\pi \left(kx+\omega t\right)}dxdt$$

where k is the wave number, and the transformed data $F\left(k,\omega \right)$ are a set of complex numbers. The maximum power of the data ${\left|F\left(k,\omega \right)\right|}^{2}$ was easily defined, and the velocity of leaky Rayleigh waves was calculated based on the ratio of the wave number and frequency. Examples of velocity calculations at different operation times are presented in Figure 10, which shows that the velocity keeps increasing as the mortar specimens harden.

**Figure 11:**
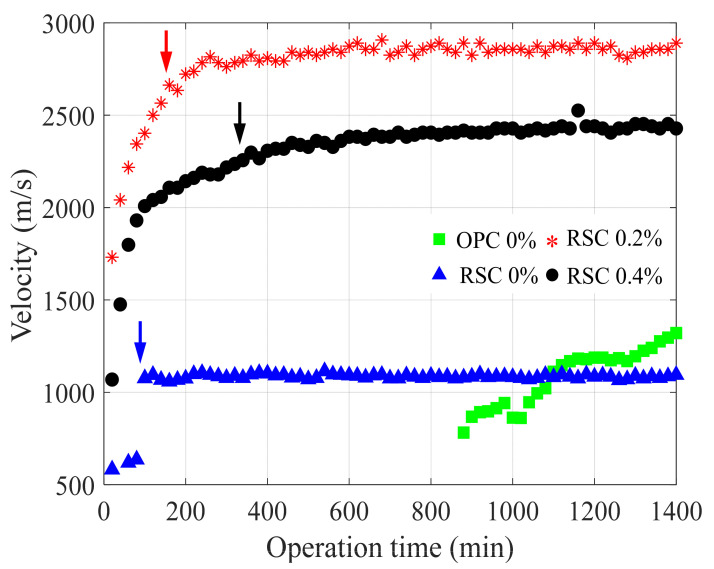
Hardening behavior monitoring of specimens in the development of leaky Rayleigh wave velocity.

Using the proposed signal processing scheme, the velocity information during operation time was identified. The velocity variation at each specimen is presented in Figure 11, where 90% of the maximum velocity over 24 h (converging point) is marked for the RSC specimens. In the case of RSC 0%, the velocity quickly reached a maximum (approximately 100 min), which is a typical of rapid-setting behavior. In contrast to RSC 0%, the velocity of OPC 0% continued to increase after the initiation of waves, and surpassed that of RSC 0% at approximately 18 h. This implies that the strength of RSC 0% was not fully developed, owing to the rapid set, unlike OPC 0%.

When the retarder was added, different hardening behaviors of the RSC specimens were identified. First, the maximum velocities of RSC 0.2% and RSC 0.4% were significantly higher than those of RSC 0%. This implies that the retarder effectively delayed the hydration progress in cementitious materials, and the rapid set did not occur. Second, the converging points were delayed when the velocity converged to its maximum, at approximately 160 and 360 min for RSC 0.2% and RSC 0.4%, respectively. The delaying converging point exhibited the residual possibility of hydration in the materials. As the retarder influences the dormant period in the hydration process, more usage of the retarder does not affect the degree of reaction but delays the beginning of the acceleration period. This corresponds to the results in which the converging point was further delayed at RSC 0.4%, although the maximum velocity was higher at RSC 0.2%. The data obtained by the sensor array enable the evaluation of unique hardening behaviors in RSC specimens, which is limited when using a single sensor.

## 5. Discussion

The proposed contactless ultrasonic method has several advantages for monitoring the hardening behavior of cementitious materials. From the experimental validation, the information of setting and hardening was identified without physical contact with the surface of the specimen. This has considerable potential for field applications because the sampling procedure is not required, and the measurement minimally interrupts the other field works. In addition, the overall procedure of data analysis can be automated.

From the perspective of civil engineering, the velocity increment during operation time can be back-calculated as the modulus of elasticity, using the relation of wave propagation and material properties. Based on Equation (4), the dynamic modulus of elasticity ($E_{d}$) can be converted from velocity. The density of the materials is easily measurable, and the Poisson’s ratio was assumed as 0.2 for general cementitious materials. The static modulus of elasticity ($E_{s}$) was further analyzed based on two theoretical models, including Popovics’ [20] as:

(10) $$E_{s}=\frac{427.52E_{d}}{\rho }$$

and Lydon and Balendran’s [21] as,

(11) $$E_{s}=0.83E_{d}$$

**Figure 12:**
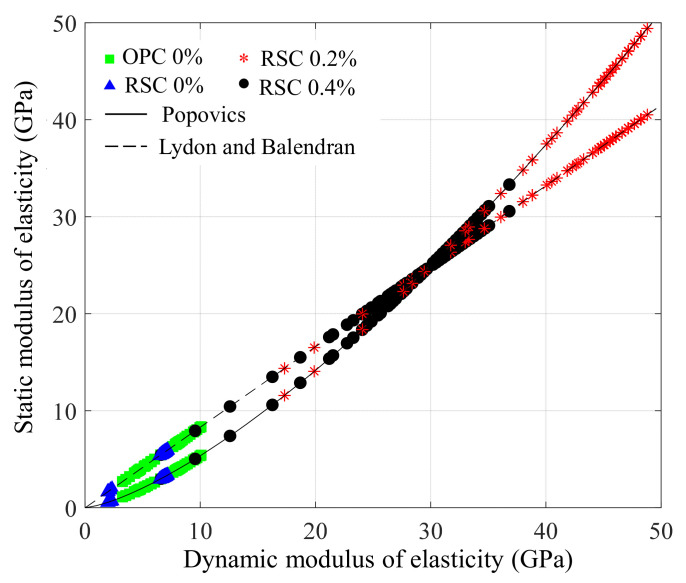
Relation between dynamic and static modulus of elasticity results converted from leaky-Rayleigh waves velocity.

**Figure 13:**
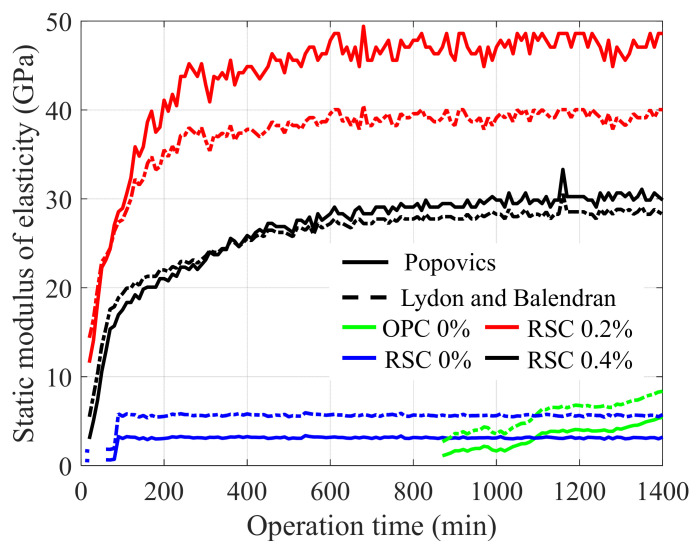
Results of hardening behavior monitoring presented as static modulus of elasticity.

Figure 12 shows the relationship between the dynamic and static modulus of elasticity by the two models, and our experimental data measured over 24 h were added to the theoretical lines. Finally, the monitoring results presented in the static modulus of elasticity for all the specimens are shown in Figure 13. The moduli are presented in the range suggested by the two models. In the case of RSC 0.4%, the range does not vary as considerably as the one in the other specimens because the modulus was developed around the crossing point (30 GPa) between the two models. Based on these results, it is confirmed that the RSC mortar produces an elastic modulus of approximately 40–50 GPa within 24 h if the proper dosage of retarder is added. Compared to the modulus of fully hardened OPC paste, known as 30 GPa [22], the RSC is demonstrated to be an effective repair material. At the same time, the use of a retarder is an important factor in RSC for delaying setting, and also for modulus development during the hydration reaction.

## 6. Conclusions

In this study, a contactless ultrasonic method was applied to monitor the setting and hardening behaviors of cementitious materials. Two different cementitious materials, RSC and OPC, were evaluated. The conclusions of this study are as follows:

1. A sensor array with an amplifying circuit was developed, and the contactless ultrasonic method clearly obtained the leakage portion of Rayleigh waves from the interface between the air and fresh mortar;
2. The RSC testing results of a single sensor show that the initiation of leaky Rayleigh waves was identified within 20 min after the addition of water, which was more than 400 times faster than that of OPC;
3. The phase differences among the signals obtained by the sensor array were calculated using a two-dimensional Fourier transform, and the velocity of leaky Rayleigh waves was accurately obtained without a subjective decision;
4. The increment of leaky Rayleigh waves from quasi-state mortar shows that the unique behaviors of hardening, such as rapid setting and the effect of retarder, were identified from RSC specimens;
5. The application of the contactless ultrasonic method was further discussed, and the static and dynamic moduli of elasticity were presented within a certain range based on the theoretical models. For further analysis of moduli in concrete, a modification of sensors will be performed to measure broadband frequencies.

## Figures and Tables

**Table 1:** Chemical components of cements utilized.

Components (% by Weight)	SiO_2_	Al_2_O_3_	Fe_2_O_3_	CaO	MgO	K_2_O	Na_2_O	SO_3_	TiO_2_	Cl
**OPC**	21.6	5.7	3.1	61.9	2.3	0.7	0.1	2.2	-	-
**RSC**	18.5	16.1	4.1	44.7	2.5	1.1	0.4	11.2	0.8	0.1

**Table 2:** Mix proportions of mortar specimens.

Specimen	W/C Ratio	Cement (kg/m^3^)	Water (kg/m^3^)	Fine Aggregate (kg/m^3^)	Setting Retarder (%)
**OPC 0%**	0.5	600	300	1200	0
**RSC 0%**					0
**RSC 0.2%**					0.2
**RSC 0.4%**					0.4

**Table 3:** Amplifying circuit parameters.

Categories	Circuit Parameters	Values
1st filter	C1	2.2 nF
	R1	510 Ω
2nd filter and 1st amplifier	C2	100 pF
	R2	4.7 kΩ
	R3	100 kΩ
Remove DC offset 1	C3	200 nF
2nd amplifier	R4	1 kΩ
	R5	100 kΩ
Remove DC offset 2	C4	10 pF
	R6	20 kΩ

## Data Availability

The data presented in this study are available upon request from the corresponding author.
